# Severe Spontaneous Hematomas in Patients Hospitalized with COVID-19

**DOI:** 10.1155/2023/6668475

**Published:** 2023-07-25

**Authors:** Silvia Otero-Rodriguez, Cristina Guillen, Maria Mataix, Pilar Gonzalez-de-la-Aleja, Elisabeth Cruces-Fuentes, Alix Juliette Mantilla-Pinilla, Oscar Moreno-Perez, Rosario Sanchez-Martinez, Esperanza Merino, Jose-Manuel Ramos-Rincon

**Affiliations:** ^1^Infectious Diseases Unit, Alicante General University Hospital-Alicante Institute of Sanitary and Biomedical Research (ISABIAL), Alicante, Spain; ^2^Endocrinology Department, Alicante General University Hospital-Alicante Institute of Sanitary and Biomedical Research (ISABIAL), Alicante, Spain; ^3^Intensive Care Unit, Alicante General University Hospital-Alicante Institute of Sanitary and Biomedical Research (ISABIAL), Alicante, Spain; ^4^Radiodiagnosis Department Alicante General University Hospital-Alicante Institute of Health and Biomedical Research (ISABIAL), Alicante, Spain; ^5^Clinical Medicine Department, Miguel Hernández University of Elche, Elche, Spain; ^6^Internal Medicine Department, Alicante General University Hospital-Alicante Institute of Health and Biomedical Research (ISABIAL), Alicante, Spain

## Abstract

**Objective:**

To describe the epidemiological, clinical, laboratory, and radiological characteristics, medical treatment, and outcomes of a case series of severe spontaneous hematoma in COVID-19. *Material and Methods*. This retrospective study included patients hospitalized for COVID-19 who were diagnosed with severe spontaneous bleeding complications by following a standardized treatment protocol that included computed tomography angiography (CTA) from 1 March 2020 to 28 February 2022. The main outcomes were embolization and all-cause mortality. Baseline variables were analyzed for their association with mortality using bivariable logistic regression, and results were expressed as odds ratios (OR) and 95% confidence intervals (CI).

**Results:**

In total, 2450 adults were hospitalized for COVID-19 in our center during the study period. 20 patients presented severe and spontaneous intramuscular bleeding (8.1 per 1000 COVID-19 admission vs. 0.47 per 1000 non-COVID-19 admissions, *p* < 0.001); their median age was 68.5 years (interquartile range (IQR) 63, 80), they had high comorbidity (median Charlson comorbidity index 4.5), and 95% were receiving high doses of heparin. The median interval from COVID-19 symptoms to bleeding was 17 days (IQR 13, 24), and 70% reported cough as a previous symptom. Hypovolemic shock, hypotension, and abdominal pain were the most frequent symptoms of the hematoma. All presented decreased hemoglobin, and 95% required transfusion. Intramuscular hematoma occurred most frequently in the rectus sheath, iliopsoas compartment, and femoral-iliac compartment. All patients underwent embolization; mortality was 45%. We did not identify risk factors associated with an increased risk of death.

**Conclusion:**

Although severe bleeding is an uncommon complication of COVID-19, its prevalence is higher than in inpatients without COVID-19, it usually needs embolization, and it is associated with high mortality.

## 1. Introduction

SARS-CoV-2 infection initially causes a hemostatic disorder with a hypercoagulable state, mainly in severe cases [1], suggesting the need for enhanced thromboprophylactic strategies that have been included in most treatment protocols since the beginning of the pandemic [2]. As inflammation starts to improve after the second week, fibrinogen and D-dimer levels decrease, which may increase bleeding risk through an unknown mechanism [3, 4]. This, combined with other risk factors for bleeding, such as cough or kidney failure, could lead to an increased incidence of hematomas in COVID-19 inpatients [4–6].

The severity of hematomas can be suspected clinically, but confirmation requires computed CTA [5–7]. This procedure sheds light on the size, location, extension, and artery responsible for the bleeding and assesses the need for treatment, also in cases of active bleeding as low as 0.3 mL/min [5–12], excluding other acute abdominal diseases and being considered the gold standard for the identification of spontaneous intramuscular hematomas over other imaging techniques [12].

The primary aim of this study is to describe incidence, clinical characteristics, and main outcomes in hospitalized COVID-19 patients with severe spontaneous hematomas diagnosed by CTA. The secondary aim is to identify factors associated with mortality.

## 2. Material and Methods

### 2.1. Study Design and Setting

A single-center retrospective study in COVID-19 patients with severe spontaneous hematomas was performed at the Alicante General University Hospital (Spain), a tertiary care institution.

Inclusion criteria were as follows: adults (≥18 years); hospitalized for community or nosocomial SARS-CoV-2 infection from 1 March 2020 to 28 February 2022; developed major spontaneous bleeding in a critical organ; with a decrease in hemoglobin of ≥2 g/dL, a requirement for ≥2 units of packed red blood cells, or presenting hypotension, hemorrhagic shock, or death [13]; and diagnosed using CTA.

#### 2.1.1. Explanatory Variables

Electronic medical records were reviewed to collect data on demographics, clinical variables (presence of acute abdominal pain or distension), intensive care unit (ICU) admission, surgical or endovascular intervention notes, and the interval between admission and imaging. Laboratory data within 3 days prior to the CTA were considered, including D-dimers, hemoglobin, fibrinogen, partial thromboplastin time, and prothrombin time. A high dose of heparin was defined as over 1 mg/kg enoxaparin every 12 h or equivalent by glomerular filtrate, whereas an intermediate dose was defined as 1 mg/kg enoxaparin every 24 h. In case another low molecular heparin was used, its equivalence with enoxaparin was calculated for an easier comparation.

#### 2.1.2. Outcome Variables

We evaluated differences in the incidence of CTA-diagnosed bleeding disorders in inpatients with versus without COVID-19 during the study period. The main outcomes were the need for embolization or blood transfusion and in-hospital all-cause mortality.

### 2.2. Image Acquisition and Analysis

All scans were performed on a 128-slice multidetector CT scanner (SOMATOM Definition Edge, Siemens, Germany), with acquisition of noncontrast images followed by acquisition during the arterial and portal venous phases. Bolus tracking was used to optimize the acquisition phase (enhancement threshold of 150 Hounsfield units). CTA detected active bleeding in all cases, showing hematoma volume and clinical severity.

Two abdominal radiologists with 10 and 15 years of experience retrospectively interpreted all CTA or computed tomography venography studies of the abdomen and pelvis in a clinical setting, assessing the site and extent of hemorrhage and the presence of active contrast extravasation.

### 2.3. Statistical Analysis

A descriptive statistical analysis was conducted, with categorical variables presented as counts and percentages and continuous variables as median and IQR. The incidence of bleeding in patients with versus without COVID-19 was compared using the chi-squared test. Baseline variables were analyzed by bivariable logistic regression to test the association with a fatal outcome, with results expressed as OR and 95% CI. Finally, a logistic regression analysis was undertaken to evaluate the association between length of hospital and ICU stay and death outcome. All analyses were performed using SPSS v25.

### 2.4. Ethical Aspects

The institutional review board approved the study. As it was retrospective, the requirement for informed consent from participants was waived (EXP. 200145). The research was conducted according to the principles of the Declaration of Helsinki.

## 3. Results

### 3.1. Patient Characteristics

In total, 2450 adults who tested positive for SARS-CoV-2 were admitted to our institution during the study period. Of all of them, 20 presented severe and spontaneous intramuscular hematoma during hospitalization. Among the COVID-19 patients with hematoma, 11 were women (55%) and 9 men (45%) with a median age of 68.5 years (IQR 63, 80) and high comorbidity (median Charlson comorbidity index 4.5). The median interval from initial COVID-19 symptoms to bleeding was 17 days (IQR 13, 24). At the time of diagnosis, 12 patients (60%) had X-ray infiltrates with over 50% opacity; 14 (70%) patients needed high-flow oxygen therapy; 10 (50%) needed invasive mechanical ventilation; and 4 (20%) were tracheostomized. Regarding medical treatment, most had been treated with corticosteroids (*n* = 17, 85%) and tocilizumab (*n* = 12, 60%).

In terms of clinical presentation, 14 (70%) patients reported coughing prior to the hematoma. The most common clinical presentation was hypovolemic shock (*n* = 16, 80%). Other common clinical findings were hypotension (*n* = 11 (55%), abdominal pain (*n* = 9; 45%), abdominal distension (*n* = 7, 35%), and palpable abdominal mass (*n* = 5, 25%). All patients presented a decreased hemoglobin level, with a median reduction of −2.7 g/dL (IQR −2.0, −5.8). We also observed a decrease in the median levels of fibrinogen (−241 mg/dL, IQR −43, −475) and D-dimers (−4.05 mg/dL, IQR −0.40, −4.08) relative to the levels recorded before the bleeding.

12 patients (60%) required ICU admission, and 8 were treated in the medical ward. Demographic, clinical, laboratory, and radiological findings, treatment, and outcome in the 20 hospitalized COVID-19 patients are shown in Table 1.

### 3.2. Use of Anticoagulation and Indications

All patients with hematoma were under treatment with heparin during hospitalization, and all but 1 of these received high doses. The main indications for anticoagulants were confirmed (*n* = 5, 24%) or probable pulmonary embolism (*n* = 4, 20%) and atrial fibrillation (*n* = 8, 40%).

### 3.3. Imaging Findings and Radiological Approach

**Figure 1:**
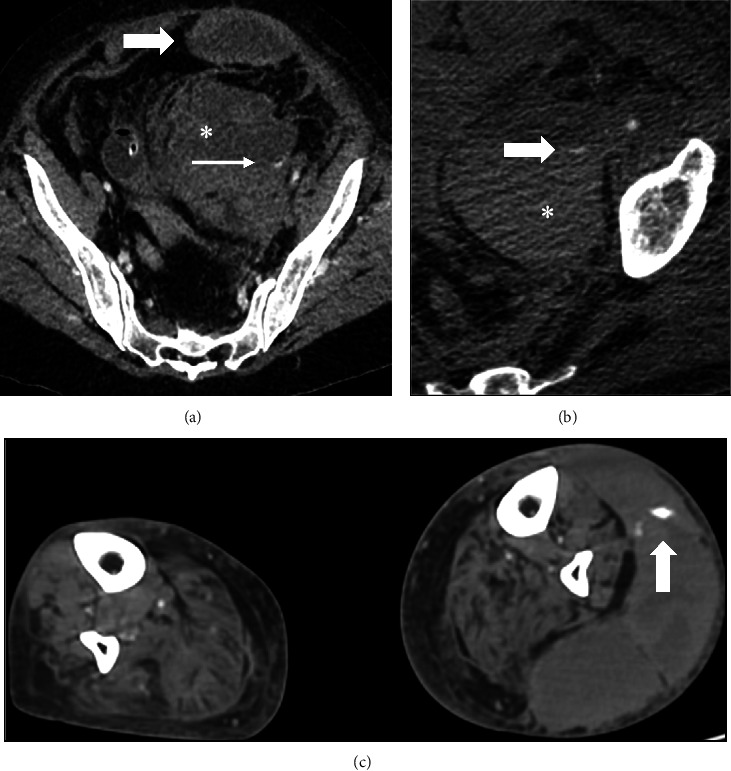
Angio-CT images of three exampled patients. (a) Left rectus sheath haematoma (gross arrow) with preperitoneal space extension (^*∗*^) and extravasation of contrast material (thin arrow). (b) Left retroperitoneal haematoma (^*∗*^) with extravasation of contrast material (arrow). (c) Left leg hematoma with active bleeding (arrow).

Intramuscular hematoma appeared in the rectus sheath (*n* = 10, 50%), iliopsoas compartment (*n* = 4, 20%), femoral-iliac compartment (*n* = 4, 20%), arm compartment (*n* = 1, 5%), and leg (*n* = 1, 5%).  Figure 1 shows angio-CT images of 3 example patients.

### 3.4. Outcomes

There were 20 cases of CTA-diagnosed bleeding disorders associated with COVID-19, for an incidence of 8.1 per 1000 admissions. During the same period, 8500 adults without COVID-19 were admitted; 4 were diagnosed with bleeding disorders via CTA (0.47 per 1000 admissions; *p* < 0.001).

**Figure 2:**
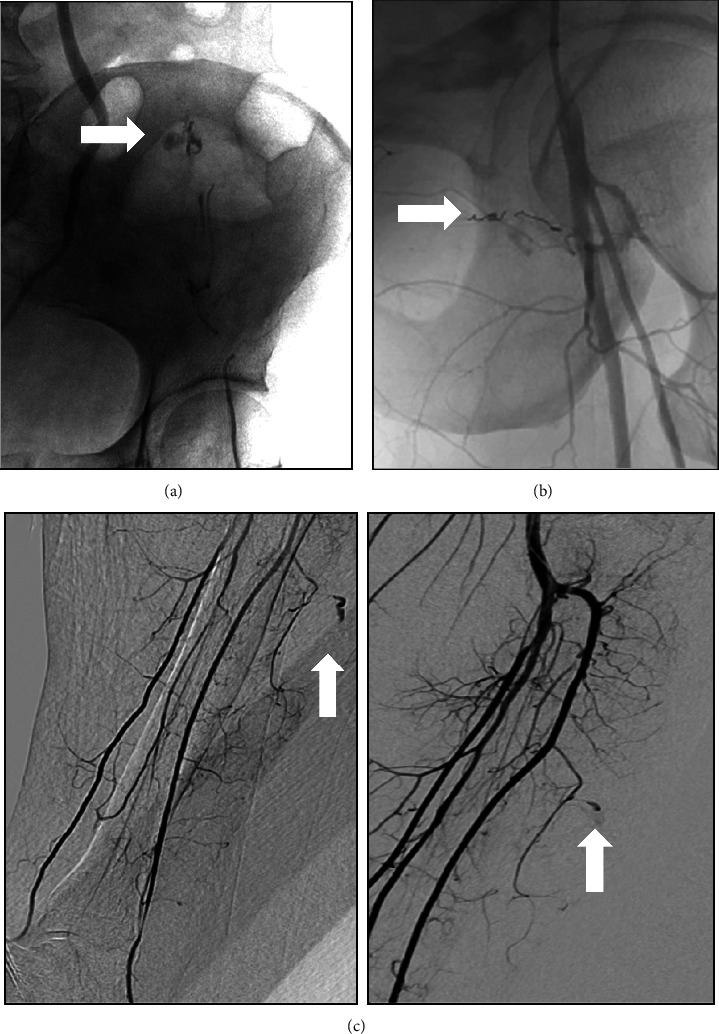
Arteriography and angioembolization of previous exampled patients. (a) Arteriography and angioembolization of the left inferior epigastric artery with liquid embolic agent. (b) Arteriography and angioembolization of a branch of the superficial femoral artery with microcoils. (c) Arteriography with active bleeding from two branches of the anterior tibial artery (arrows), embolized with coils and glue.

All patients underwent embolization, and 1 patient needed hematoma drainage. The main vessel involved in hemorrhagic complications was the epigastric artery (*n* = 9; 45%), followed by the lumbar (*n* = 4, 20%) and femoral (*n* = 3, 15.0%) arteries. The hypogastric, iliac, subclavian, and tibial arteries were involved in 1 patient each.  Figure 2 shows arteriography and angioembolization of previous examples of patients.

Nineteen patients (95%) received a blood transfusion, receiving a median 4 units (IQR 2.25, 4.75). The anticoagulant treatment was stopped in 15 patients (75%) and reduced in the other 5 (25%). Moreover, all patients needed hemodynamic support with intravenous fluid.

Finally, 9 patients (45%) died; the cause of death was indirectly related to the hemorrhagic complication in 5 (24%) and directly related in 4 (20%). The bivariable logistic regression analysis did not show evidence that any risk factors (epidemiological data, comorbidities, clinical, laboratory, and treatment) were associated with a fatal outcome among those with hemorrhagic bleeding (Table 2).

### 3.5. Other Results

We also describe the general COVID-19 cohort without hematoma (*N* = 2430). Among them, 1108 (45.6%) were women and 1321 (54.4%) men, with a median age of 63.1 years (IQR 52, 76) and the median Charlson comorbidity index of 3.1. At the time of diagnosis, 605 (24.9%) patients had X-ray infiltrates with over 50% opacity, 464 (19.1%) needed high-flow oxygen therapy, and 267 (11%) needed invasive mechanical ventilation. Regarding medical treatment, 1285 (52.9%) received prophylactic heparin, 736 (30.3%) were treated with corticosteroids, and 559 (23%) with tocilizumab. 413 patients (17%) required ICU admission. 240 patients (9.9%) died.

Among the non-COVID-19 patients with hematoma (*N* = 4), all of them were men, with a median age of 72 (IQR 70, 76) and a median Charlson comorbidity index of 4.5. 1/4 (25%) had corticoid treatment, and 3/4 were treated with high-dose heparin when bleeding was diagnosed. 2/4 (50%) had hemorrhagic shock and needed ICU admission, without any death.

## 4. Discussion

In this series of 20 cases of spontaneous hematomas in COVID-19 patients, major bleeding was both a quantitatively relevant and clinically severe complication (8.1 per 1000 COVID-19 admissions, with high direct and indirect mortality (45%)). The incidence was significantly higher in patients with COVID-19 compared to those without. The diagnosis was confirmed using CTA, and all patients underwent embolization.

The prevalence of severe spontaneous hemorrhage in hospitalized patients with COVID-19 is not well described in the medical literature. Sposato et al. [11] showed a prevalence of 1.3%, whereas Mahmoudabadi et al.[14] reported that 0.4% of hospitalized patients with COVID-19 developed a rectus sheath hematoma. In our study, the prevalence of spontaneous hematoma was 0.8%, the midpoint between these other two studies.

The patient profile in our series was characterized by having a high number of comorbidities and being under treatment with a therapeutic dose of anticoagulants due to the diagnosis or suspicion of pulmonary embolism or atrial fibrillation, which is consistent with previous reports [5–8, 11, 14, 15]. Another important risk factor related to spontaneous bleeding was cough, a common sign in these patients before the event. In some studies, bleeding has also been reported to be more common in elderly patients [15]. In our case, 15% were over 80 years of age. Hypotension, with or without hypovolemic shock, was a common clinical situation in these patients, and acute anemia was a typical finding in the blood tests [5–8, 11, 14, 15]. Regarding the respiratory status, most of the patients that bled had a moderate-to-severe COVID-19 presentation (60% with more than 50% opacities in X-ray and up to 70% with high-flow oxygen therapy need), in contrast with the general nonbleeding COVID-19 cohort (24.9% and 19.1% respectively), which could influence the higher corticosteroids dose found in the bleeding cohort (85% vs. 30.3%). This leads us to think about giving special importance to risk assessment and early signs of bleeding in this kind of patient and carrying out an adapted management in COVID-19 protocols, which until now more focused on prothrombotic risk (for instance, early surveillance of wall hematomas, detection of added bleeding factors, and an early CTA scan in those with hemorrhagic shock or acute abdominal pain).

The most common sites of bleeding reported in the literature are the rectus sheath, iliopsoas compartment, and retroperitoneal space [5–8, 11, 14, 15], in keeping with our results. Given the severity of this entity, even with prompt suspicion, diagnosis, and appropriate management with supportive treatment (including fluids and blood transfusion), endovascular treatment for transarterial embolization at the origin was necessary in all our cases. This is also common in most patients described in the literature [5–8, 12, 15].

The mortality in our study was slightly under 50%, while in other studies it has surpassed the 50% mark [5, 6, 8, 14, 15]. Our results may be explained by the effectiveness of the endovascular treatment as a life-saving procedure. In general, the mortality described in COVID-19 patients is higher than in those without COVID-19, which is about 25% [12]. This could be related to the pathophysiology of COVID-19 or the associated severe respiratory insufficiency [5, 6, 15].

We have tried to identify the risk factors associated with worse outcomes in patients with spontaneous hematoma in order to contribute to earlier detection and optimal management. However, none of the factors analyzed were significantly associated with increased mortality, including age, comorbidities, or persistent cough—all well-known risk factors for spontaneous hematoma [5, 15]. Regarding analytical data, some research evidence supports the involvement of inflammation markers like fibrinogen in bleeding severity, as a decline in fibrinogen and D-dimers concentration has been described between the second and third weeks and an average of 4 days before bleeding starts [3, 4]. We did not observe differences in laboratory data, although the median interval from initial COVID-19 symptoms to bleeding (17 days) fell within the range described. A rigorous systematic review and meta-analysis could probably resolve the uncertainties about these variables as risk factors for an unfavorable outcome.

The strengths of this study include the high number of cases and the standardized treatment protocol. However, there are also some limitations. Firstly, electronic medical records were evaluated retrospectively, which may have resulted in an information bias (a large number of laboratory variables were missing, which could have influenced our results). Secondly, this is a single-center study, so caution is warranted when extrapolating results to other healthcare settings. Thirdly, the pathological correlation with imaging abnormalities was not available for our patients. Finally, inclusion criteria were limited to major bleeding, so we may have underestimated the real prevalence of bleeding events in COVID-19 patients.

## 5. Conclusion

Although severe bleeding is an uncommon complication in patients with COVID-19, it is more frequent than in patients with other conditions. Moreover, patients hospitalized for COVID-19 frequently take anticoagulants that could make bleeding easier, and they may have other risk factors that increase the risk of hemorrhage, although a bivariable logistic regression did not reveal any that were predictors of mortality. Embolization techniques are usually needed, but even with effective treatment, spontaneous hematomas entail a high risk of death.

## Figures and Tables

**Table 1:** Demographic, clinical, laboratory, and radiological findings, treatment, and outcome in 20 hospitalized COVID-19 patients with severe spontaneous hematoma.

Variables		*N* = 20
*Demographics*		
Age, years, median (IQR)		68.5 (63.3–79.8)
Men, *n* (%)		9 (45)
Length of stay before diagnosis, days, median (IQR)		12 (7.3–19.5)
Interval from initial symptoms to bleeding, days, median (IQR)		17 (13–24)
Full SARS-CoV-2 vaccination, *n* (%)		6 (30.0)

*SARS-CoV-2 strain, n (%)*		
Wuhan		3 (15.0)
Alpha/Beta		8 (40.0)
Delta		1 (5.0)
Omicron		8 (40.0)

*Comorbidities, n (%)* ^*∗*^		
Charlson comorbidity index, median (IQR)		4.5 (2.0–7.0)
Hypertension		12 (60.0)
Dyslipidemia		11 (55.0)
Smoking		8 (44.0)
Chronic kidney disease		9 (45.0)
Obesity		9 (45.0)
Diabetes		6 (30.0)
Chronic obstructive pulmonary disease		4 (20.0)
Immunosuppression		6 (30.0)
Auricular fibrillation		5 (25.0)
Chronic heart failure		5 (25.0)
Ischemic cardiopathy		4 (20.0)
Neoplasia		2 (10.0)
Rheumatoid arthritis		2 (10.0)
Arteriopathy		2 (10.0)
Systemic erythematosus lupus		1 (8.0)

*Symptoms at diagnosis of spontaneous hematoma, n (%)*		
Cough		14 (70.0)
Abdominal pain		9 (45.0)
Abdominal distension		7 (35.0)
Palpable abdominal mass		5 (25.0)
Visible hematoma		4 (20.0)

*Physical examination at diagnosis of spontaneous hematoma, n (%)*		
Hypovolemic shock		16 (80.0)
Hypotension		11 (55.0)

*Analytical results, median (IQR)*		
Hemoglobin, g/dL	Baseline	11.0 (9.4, 12.6)
	After diagnosis of hematoma	7.3 (6.5, 7.9)
	Difference in medians	−2.7 (−2.0, −5.8)
Fibrinogen, mg/dL	Baseline	602 (541, 783)
	After diagnosis of hematoma	352 (197, 523)
	Difference in medians	−241 (−43, −475)
D-dimers, mg/mL	Baseline	4.38 (1.30, 14.30)
	After diagnosis of hematoma	1.66 (0.96, 6.71)
	Difference in medians	−4.05 (−0.40, −4.08)
Partial thromboplastin time at diagnosis, seconds		1.11 (0.90, 1.4)

*Ventilatory situation at diagnosis of spontaneous hematoma, n (%)*		
High-flow oxygen therapy		14 (70.0)
Invasive mechanical ventilation		10 (50.0)
Tracheostomy		4 (20.0)

Medical treatment for COVID-19, *n* (%)		
Corticosteroids		17 (85.0)
Dexamethasone, 6 mg every 24 hours		12 (60.0)
Methylprednisolone 40 mg every 12 hours		5 (25.0)
Tocilizumab		12 (60.0)
Plasma		5 (25.0)
Remdesivir		8 (40.0)

*Anticoagulant therapy*		
High dose		19 (95.0)
Intermediate dose		1 (5.0)
Prophylaxis		0 (0.0)

*Indication for anticoagulant therapy*		
Atrial fibrillation		8 (40.0)
Pulmonary embolism, confirmed		5 (24.0)
Pulmonary embolism, probable		4 (20.0)
Mechanical valvular prothesis		2 (10.0)
Lupus anticoagulant		1 (5.0)
Prophylaxis		1 (5.0)

*Location of hematoma identified by CTA*		
Rectus sheath		10 (50.0)
Iliopsoas compartment		4 (20.0)
Femoral-iliac compartment		4 (20.0)
Arm compartment		1 (5.0)
Leg compartment		1 (5.0)

*X-ray at diagnosis* ^*∗∗*^		
Opacity >50%		12 (60.0)
Embolization		20 (100)

*Artery of embolization*		
Epigastric		9 (45.0)
Lumbar		4 (20.0)
Femoral		3 (15.09)
Hypogastric		1 (5.0)
Iliac		1 (5.0)
Subclavian		1 (5.0)
Tibial		1 (5.0)

*Treatment*		
Drainage of hematoma		1 (5.0)
Blood transfusion		19 (95.0)
Units of blood transfusion, median (IQR)		4 (2.25–4.75)
Discontinuation of heparins		15 (75.0)

*Characteristics of admission during hematoma*		
Intensive care unit		12 (60.0)
Length of ICU stay, days, median (IQR)		25 (3–90)

*Outcome*		
Length of total stay, days, median (IQR)		33.5 (20–75)
Death, all causes		9 (45.0)
Death directly related to hematoma		4 (20.0)
Death indirectly related to hematoma		5 (24.0)

IQR: interquartile range; CTA, computerized tomography angiography. ^*∗*^Except where otherwise noted; ^*∗∗*^at diagnosis of spontaneous hematoma.

**Table 2:** Results of bivariable logistic regression for risk factors associated with fatal outcome.

		Died (*n* = 9)	Survived (*n* = 11)	Or (95% CI)
*Demographics*				
Age, years, median (IQR)		68 (65–73)	69 (62.5–79.5)	1.01 (0.92, 1.12)
Men, *n* (%)		3 (33.3)	6 (54.5)	0.42 (0.67, 2.58)

*Comorbidities, n (%)* ^*∗*^				
Charlson comorbidity index, median (IQR)		3 (3–6)	6 (2–7)	0.96 (0.70, 1.30)
Hypertension		4 (44.4)	8 (72.7)	0.30 (0.05, 1.94)
Dyslipidemia		6 (66.7)	5 (45.5)	2.40 (0.39, 14.88)
Smoking		5 (55.6)	3 (27.3)	3.33 (0.52, 21.58)
Chronic kidney disease		5 (55.6)	4 (36.4)	2.19 (0.36, 13.23)
Obesity		3(33.3)	6 (54.5)	0.42 (0.07, 2.58)
Diabetes		3 (33.3)	3 (27.3)	1.33 (0.20, 9.08)
Respiratory disease		4 (44.4)	0 (0.0)	1.25 (0.07, 23.26)
Immunosuppression		3 (33.4)	3 (27.3)	1.33 (0.20, 9.08)
Auricular fibrillation		2 (22.2)	3 (27.3)	0.76 (0.10, 5.96)
Chronic heart failure		2 (22.3)	3 (27.3)	0.76 (0.10, 5.96)
Ischemic cardiopathy		3 (3.3)	1 (9.7)	5.00 (0.42, 59.66)
Arteriopathy		1 (11.1)	1 (9.19)	1.25 (0.07, 23.26)
Pulmonary thromboembolism		1 (11.1)	0 (0)	0.76 (0.10, 5.96)

*Symptoms at diagnosis of spontaneous hematoma, n (%)*				
Cough		6 (66.7)	8 (72.7)	0.75 (0.11, 5.11)
Abdominal pain		5 (55.6)	5 (36.4)	2.19 (0.36, 13.23)
Abdominal distension		4 (44.5)	3 (27.3)	2.13 (0.33, 13.81)
Palpable abdominal mass		4 (44.4)	1 (9.1)	8.00 (0.70, 91.80)
Visible hematoma		3 (33.3)	1 (9.1)	5.00 (0.42, 59.66)

*Physical examination at diagnosis of spontaneous hematoma, n (%)*				
Hypovolemic shock		7 (77.8)	9 (81.8)	0.78 (0.09, 6.98)
Hypotension		3 (33.4)	8 (72.7)	0.19 (0.03, 1.28)

*Analytical results, median (IQR)*				
Hemoglobin, g/dL	Baseline	9.7 (9.4, 11.9)	11.1 (10.0–12.5)	0.92 (0.61, 1.38)
	After hematoma diagnosis	7.5 (7.0, 7.9)	7.2 (6.4–7.7)	1.52 (0.65, 3.56)
	Difference in medians	−2.4 (−2.0, −3.3)	−4.2 (−2.2, −5.9)	0.81 (0.52, 1.27)
Fibrinogen, mg/dL	Baseline	482 (421, 600)	668 (601, 883)	0.99 (0.98, 1.00)
	After hematoma diagnosis	438 (296, 639)	276 (160, 475)	1.00 (1.00, 1.01)
	Difference in medians	−190 (32, −240)	−409 (−227, −658)	0.99 (0.99, 1.00)
D-dimers, mg/mL	Baseline	3.97 (1.4, 12.30)	4.8 (1.54, 11.32)	0.97 (0.86, 1.10)
	After hematoma diagnosis	1.70 (1.30, 10.5)	1.63 (1.06, 4.5)	1.06 (0.95, 1.19)
	Difference in medians	−4.20 (−0.41, −5.60)	−0.70 (−0.39, −5.44)	0.99 (0.97, 1.01)
Partial thromboplastin time (s) at diagnosis		1.26 (1.02, 1.45)	1.00 (0.90, 1.23)	2.43 (0.15, 40.50)

*Interventions being administered at diagnosis of spontaneous hematoma*				
High-flow oxygen therapy		8 (88.9)	6 (54.5)	6.67 (0.61, 73.03)
Invasive mechanical ventilation		5 (55.5)	5 (45.5)	1.50 (0.26, 8.82)
Tracheostomy		2 (22.2)	2 (18.2)	1.29 (0.14, 11.54)
Corticosteroids		7 (77.8)	10 (90.9)	0.35 (0.03, 4.65)

*Characteristics of admission during hematoma*				
Intensive care unit		5 (55.0)	7 (63.6)	0.71 (0.12, 4.32)
Length of ICU stay, days, median (IQR)		20 (2–30)	30 (5–95)	0.99 (0.96, 1.02)
Total length of hospital stay, days median (IQR)		24 (20–75)	39 (23–83)	0.96 (0.88, 1.05)

IQR: interquartile range; OR: odds ratio, ^*∗*^unless otherwise noted.

## Data Availability

The dataset used to support the findings of this study are restricted by the Hospital de Alicante ethics committee (EXP. 20014) in order to protect patient privacity. Data are available from J.M. Ramos-Rincon (jose.ramosr@umh.es) for researchers who meet the criteria for access to confidential data.

## Additional Points

(i) Major bleeding in COVID-19 is an uncommon complication, but more frequent than in patients admitted with other conditions. (ii) Patients had a high rate of therapeutic anticoagulation and comorbidities. (iii) The fatality rate was around 50% and embolization was usually required. (iv) When comparing conditions associated with death with bivariable logistic regression, we did not identify risk factors associated with an increased risk of death. (v) Further analysis is required to identify risk mortality factors in order to prevent bleeding.
